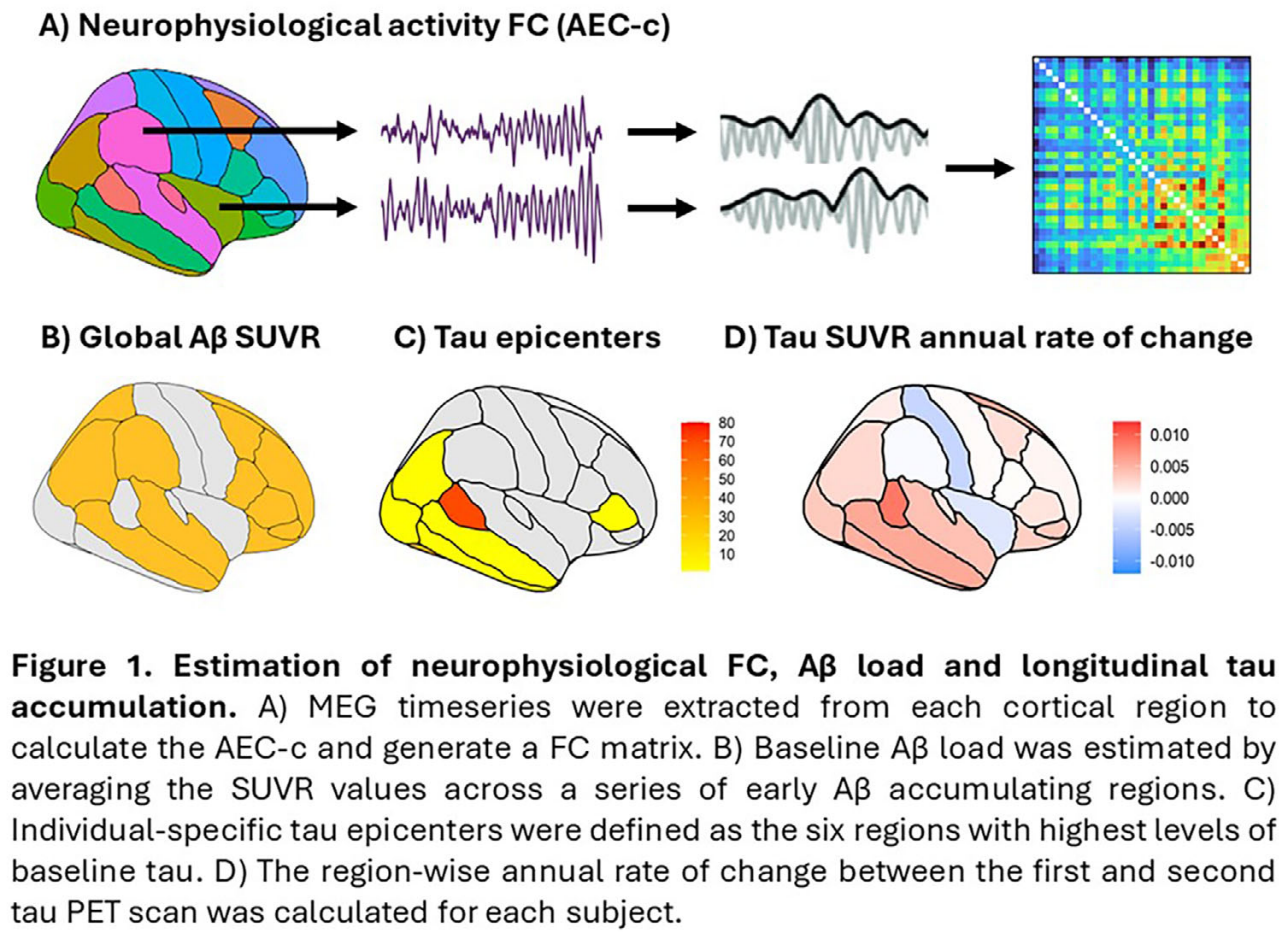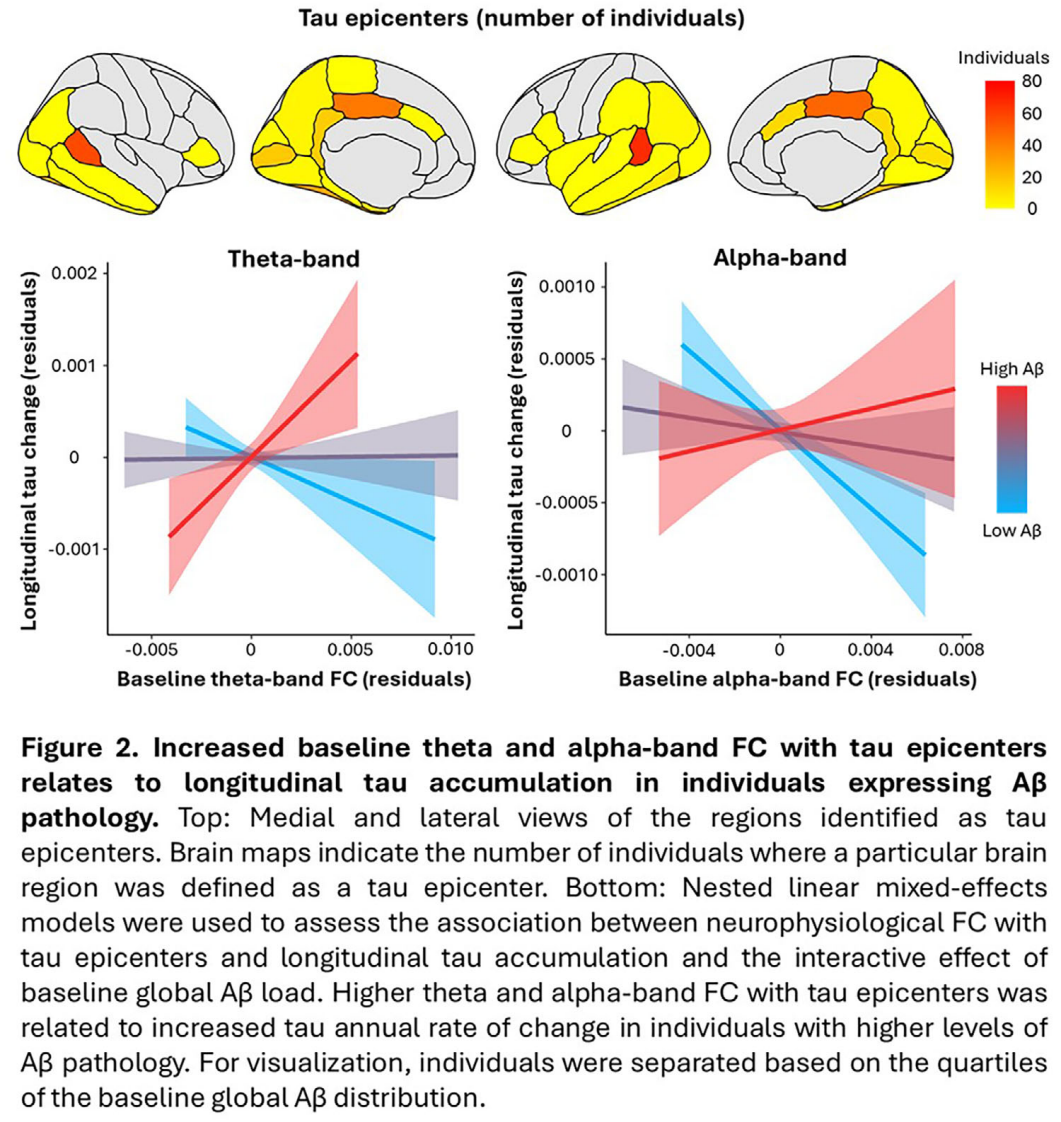# Theta‐ and alpha‐band functional connectivity are associated with longitudinal tau accumulation in asymptomatic individuals expressing Aβ pathology

**DOI:** 10.1002/alz.094010

**Published:** 2025-01-09

**Authors:** Jonathan Gallego Rudolf, Alex I Wiesman, Sylvain Baillet, Sylvia Villeneuve

**Affiliations:** ^1^ Douglas Mental Health Research Centre, Montreal, QC Canada; ^2^ Montreal Neurological Institute, Montreal, QC Canada

## Abstract

**Background:**

Recent studies suggest that tau pathology spreads between functionally connected (FC) brain regions. The accumulation of amyloid‐beta (Aß) promotes neural hyper‐activity in asymptomatic older adults, which might enhance tau spreading. We assessed the relationship between band‐specific neurophysiological FC and the rate of tau accumulation over time and tested whether this association is dependent on the presence of Aß pathology.

**Method:**

We measured cortical Aß deposition using positron emission tomography (PET) and resting‐state neurophysiological activity using magnetoencephalography (MEG) at baseline, as well as longitudinal cortical tau PET deposition (mean time between scans = 4.3 years), in a group of cognitively unimpaired older adults with family history of Alzheimer’s Disease (PREVENT‐AD cohort; N = 84). Region‐specific measures (i.e. Aß, tau, MEG timeseries) were extracted from 68 cortical parcels of the Desikan‐Killiany atlas. Neurophysiological FC between cortical regions was estimated across delta, theta, alpha and beta frequency bands using the corrected amplitude envelope correlation (AEC‐c). A global Aß standardized uptake value ratio (SUVR) was calculated by taking the average across a set of early Aß accumulating regions. Individual‐specific tau epicenters were defined as the six cortical regions with the highest levels of baseline tau‐PET binding. The annual rate of change in tau SUVR between the first and second scan was calculated for each cortical region (Figure 1). Linear mixed‐effects models were designed to test if the neurophysiological FC connectivity of each cortical parcel with tau epicenters was related to increased longitudinal tau accumulation in that region, and whether this association was dependent on baseline levels of Aß pathology.

**Result:**

Tau epicenters identified across individuals were mainly localized in temporal and medial posterior brain regions. Regional increases in theta‐ and alpha‐band FC with tau epicenters at baseline related to greater longitudinal tau accumulation in individuals expressing higher levels of global Aß pathology (Figure 2).

**Conclusion:**

Our results suggest that tau spreads across cortical regions that are functionally connected in the theta‐ and alpha‐bands, but only in individuals with high levels of cortical Aß. This finding supports the notion that Aß‐related neural hyper‐activity promotes tau spreading across the cortex following individual‐specific patterns of FC in asymptomatic older adults.